# Ubiquinone-binding site mutagenesis reveals the role of mitochondrial complex II in cell death initiation

**DOI:** 10.1038/cddis.2015.110

**Published:** 2015-05-07

**Authors:** K Kluckova, M Sticha, J Cerny, T Mracek, L Dong, Z Drahota, E Gottlieb, J Neuzil, J Rohlena

**Affiliations:** 1 Institute of Biotechnology, Academy of Sciences of the Czech Republic, Prague, Czech Republic; 2 Faculty of Sciences, Charles University, Prague, Czech Republic; 3 Institute of Physiology, Academy of Sciences of the Czech Republic, Prague, Czech Republic; 4 School of Medical Science, Griffith University, Southport, Queensland, Australia; 5 The Beatson Institute for Cancer Research, Glasgow, UK

## Abstract

Respiratory complex II (CII, succinate dehydrogenase, SDH) inhibition can induce cell death, but the mechanistic details need clarification. To elucidate the role of reactive oxygen species (ROS) formation upon the ubiquinone-binding (Q_p_) site blockade, we substituted CII subunit C (SDHC) residues lining the Q_p_ site by site-directed mutagenesis. Cell lines carrying these mutations were characterized on the bases of CII activity and exposed to Q_p_ site inhibitors MitoVES, thenoyltrifluoroacetone (TTFA) and Atpenin A5. We found that I56F and S68A SDHC variants, which support succinate-mediated respiration and maintain low intracellular succinate, were less efficiently inhibited by MitoVES than the wild-type (WT) variant. Importantly, associated ROS generation and cell death induction was also impaired, and cell death in the WT cells was malonate and catalase sensitive. In contrast, the S68A variant was much more susceptible to TTFA inhibition than the I56F variant or the WT CII, which was again reflected by enhanced ROS formation and increased malonate- and catalase-sensitive cell death induction. The R72C variant that accumulates intracellular succinate due to compromised CII activity was resistant to MitoVES and TTFA treatment and did not increase ROS, even though TTFA efficiently generated ROS at low succinate in mitochondria isolated from R72C cells. Similarly, the high-affinity Q_p_ site inhibitor Atpenin A5 rapidly increased intracellular succinate in WT cells but did not induce ROS or cell death, unlike MitoVES and TTFA that upregulated succinate only moderately. These results demonstrate that cell death initiation upon CII inhibition depends on ROS and that the extent of cell death correlates with the potency of inhibition at the Q_p_ site unless intracellular succinate is high. In addition, this validates the Q_p_ site of CII as a target for cell death induction with relevance to cancer therapy.

Mitochondrial respiratory complex II (CII), aka succinate dehydrogenase (SDH), directly links the tricarboxylic acid (TCA) cycle to the electron transport chain (ETC) by mediating electron transfer from the TCA cycle metabolite succinate to ubiquinone (UbQ).^1^ For this reason, CII is subjected to a high electron flux between the succinate-binding dicarboxylate site in the matrix-exposed subunit A and the proximal UbQ-binding (Q_p_) site, formed by the subunits C (SDHC) and D embedded in the mitochondrial inner membrane (Figure 1b).^2, 3, 4, 5^ Disruption of electron transfer to UbQ, for example by Q_p_ site inhibition, leads to reactive oxygen species (ROS) generation from CII due to the leakage of ‘stalled' electrons to molecular oxygen at the reduced flavin adenine dinucleotide (FAD) prosthetic group. However, ROS production from reduced FAD is only possible when the adjacent dicarboxylate site is neither occupied by its substrate succinate, typically at low succinate conditions, nor inhibited by other dicarboxylates, for example by malonate.^6, 7, 8, 9, 10^

Beyond bioenergetics, CII has emerged as an important factor in cell death induction.^11, 12^ On one hand, it has been proposed that increased ROS production from CII, resulting from changes in matrix pH and calcium status, amplifies cell death signals originating at other sites.^12, 13, 14, 15^ On the other hand, the inhibition of CII may also directly initiate cell death, as suggested by our previous results with vitamin E (VE) analogs such as the mitochondrially targeted VE succinate (MitoVES). This compound inhibits CII activity leading to ROS generation and cell death induction in cancer cells, as evidenced by the suppression of tumor growth in experimental animal models.^16, 17, 18, 19, 20^ The efficacy of MitoVES is greatly reduced in the absence of functional CII, and computer modeling along with other corroborative evidence suggests that MitoVES binds to the Q_p_ site of CII.^16^ However, this is only circumstantial evidence with respect to cell death induction, as cells lacking electron flux within CII due to a structural defect should not be able to produce CII-derived ROS. Accordingly, not only the direct cell death initiation upon CII inhibition will be compromised in this situation, but also the indirect signal amplification mentioned above will be affected.

In the present study, we combined site-directed mutagenesis of Q_p_ site amino-acid residues with the use of Q_p_ site inhibitors MitoVES, thenoyltrifluoroacetone (TTFA) and Atpenin A5 to assess the link between Q_p_ site inhibition and cell death initiation. We show that for MitoVES and TTFA, the potency of Q_p_ site inhibition correlates with the extent of ROS production and cell death induction in respiration-competent CII variants, and that the induced cell death is dependent on CII-derived ROS.

Atpenin, however, did not induce cell death, possibly due to the rapid accumulation of succinate in intact cells, incompatible with ROS generation from CII. These results provide evidence for the role of CII in cell death initiation and establish the Q_p_ site as a target for cell death induction.

## Results

### CII Q_p_ site mutagenesis and the experimental model

**Figure 1:**
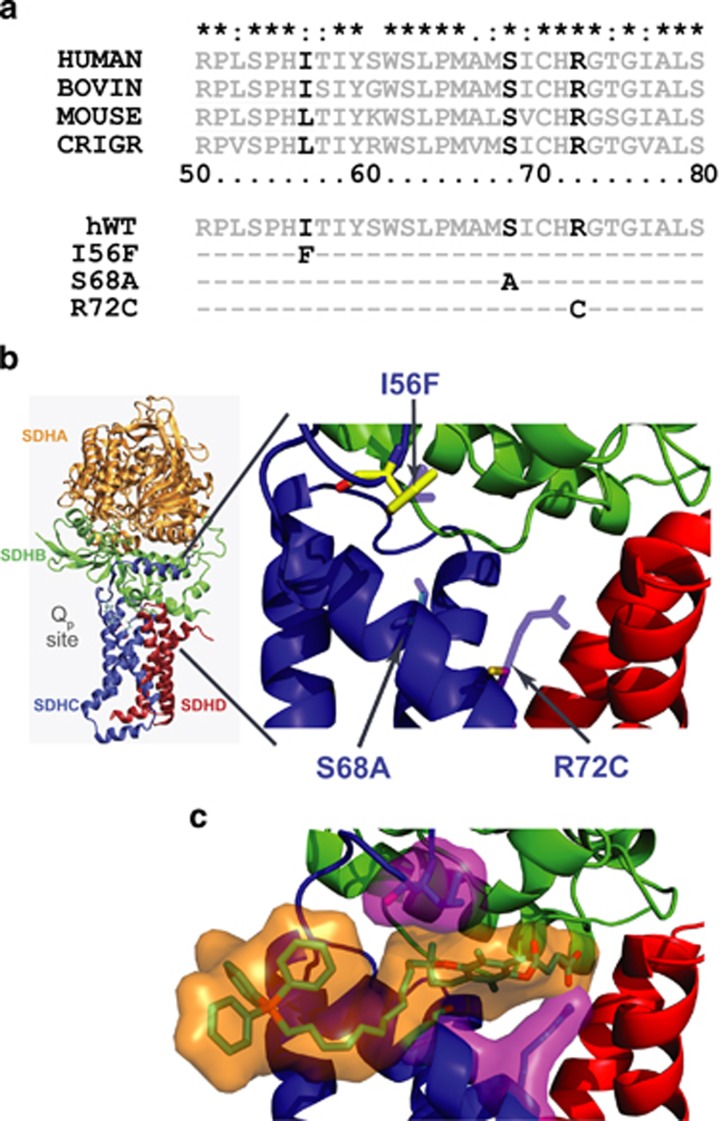
Amino-acid substitutions in the Q_p_ site of CII. (**a**) Multiple species alignment of the SDHC region bordering the Q_p_ site shows a high level of conservation. Amino-acid substitutions prepared for this study are indicated in human SDHC. (**b**) Three dimensional representation of CII and the topology of the Q_p_ site. SDHC residues mutated in this study are indicated by arrows. Displayed is the humanized crystal structure of porcine CII.^3^ (**c**) A snapshot from molecular dynamics simulation of MitoVES interaction with the Q_p_ site of CII in the presence of phospholipid bilayer.^16^ One of the possible conformations of MitoVES is shown in orange, substituted SDHC residues are depicted in magenta

To explore the role of CII in cell death induction, we performed site-directed mutagenesis within SDHC, a CII subunit that contributes to Q_p_ site formation.^3^ We concentrated on SDHC residues predicted to be in close contact with bound MitoVES. Serine 68 was mutated to alanine, arginine 72 was replaced by cysteine, and isoleucine 56 was substituted by phenylalanine (Figure 1). Recent data indicate reduced cell death induction by MitoVES in the S68A variant,^16^ but the functional consequences of this mutation for CII activity have not been studied. Nevertheless, substitutions of S68 as well as of R72 are expected to compromise CII activity, based on analogy with *E.coli* and *S. cerevisiae* SDH.^21, 22, 23, 24^ The I56 residue is further away from the Q_p_ site and its role in CII function is unknown. To evaluate these substitutions, we utilized a mammalian model of SDHC deficiency, the Chinese hamster lung fibroblast cell line B9.^25^ These cells lack the functional CII due to a nonsense mutation in the SDHC subunit and fail to assemble CII. In consequence they do not respire on succinate and are completely devoid of CII enzymatic activity. Stable transfectants of human wild-type (WT) and variant SDHC cDNA were prepared in B9 cells, and the clones with similar level of SDHC were selected. These cells were further transformed by H-Ras fused to green fluorescence protein (GFP), making them plausible models to study the effect of MitoVES and other Q_p_ site inhibitors on transformed cells (Supplementary Figure S1). Transformants with similar level of H-Ras were selected to control for H-Ras level.

### Q_p_ site mutations differentially affect CII assembly and enzymatic function

**Figure 2:**
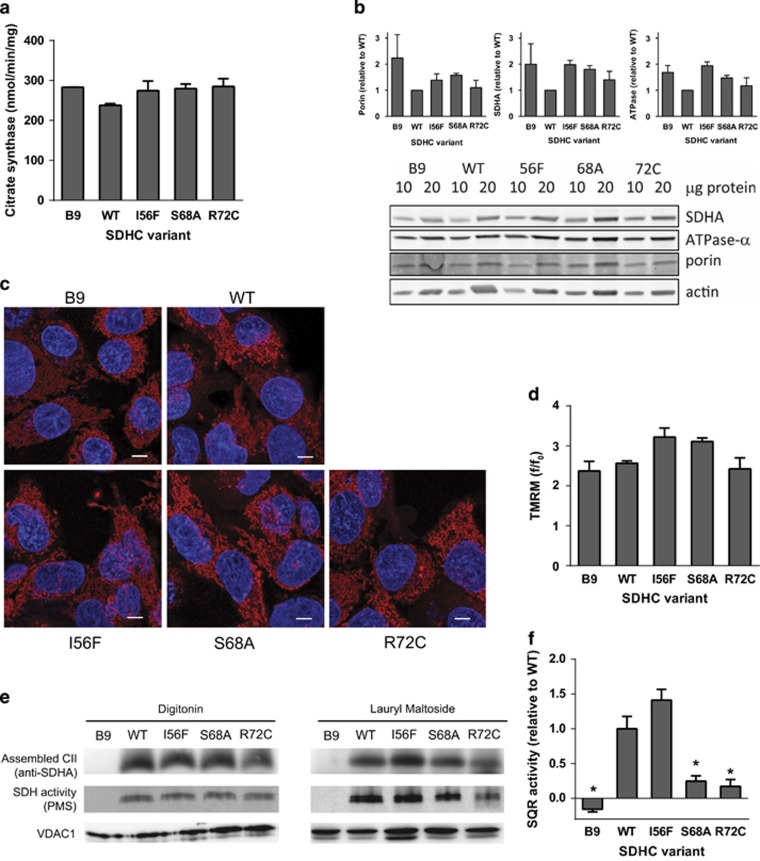
Q_p_ site substitutions do not decrease mitochondrial content, membrane potential or CII assembly, but can compromise CII activity in the presence of detergents. (**a**) Citrate synthase was measured in the whole-cell lysates and corrected to the protein content. (**b**) Selected mitochondrial proteins were analyzed by western blotting using 10 and 20 *μ*g of whole-cell protein. A representative blot is shown along with quantifications based on three independent experiments (**c**) Mitochondria were visualized by live confocal microscopy using TMRM, the nuclei were counterstained by Hoechst 33342. Scale bar, 5 *μ*m. (**d**) Mitochondrial membrane potential was determined as a ratio of TMRM loading in the presence and absence of FCCP, *n*=3, mean±S.E.M. (**e**) Native blue gel electrophoresis of either digitonin- or lauryl maltoside-solubilized mitochondrial fraction isolated from CII variant cell lines. Assembled CII was detected by anti-SDHA antibody, or by in-gel SDH activity assay using PMS (phenazine methosulfate). Representative experiments are shown (**f**) SQR activity measurement in isolated mitochondrial fraction in the presence of 0.1% Triton X-100 indicates activity impairment for amino-acid substitutions in position S68 and R72. Data represent mean values±S.E.M. of three independent experiments. The symbol * indicates values significantly different from WT

The selected clones did not significantly differ in their mitochondrial content, as evidenced by similar citrate synthase activity and mitochondrial protein levels (Figures 2a and b). Mitochondrial morphology and membrane potential were also similar for all the tested cell lines (Figures 2c and d). To verify CII assembly, mitochondrial fractions were subjected to blue native gel electrophoresis and western blotting using an anti-SDHA antibody. As expected, parental B9 cells did not assemble CII. In contrast, CII was fully assembled in WT and most of the SDHC variant cells (Figure 2e), with a minor assembly defect found for R72C variant. These results were confirmed using an in-gel SDH activity assay (Figure 2e), which documented assembled CII with functional dicarboxylate site in all variants except for B9 cells. To assess the condition of the Q_p_ site and its functional coupling to the dicarboxylate site, succinate-UbQ reductase (SQR) activity in the mitochondrial fraction was determined. Whereas no SQR activity was measurable in B9 cells (Figure 2f), it was high in WT and I56F clones. For S68A and R72C variants the SQR activity was significantly reduced yet remained above the level of parental B9 cells. This suggests that although CII assembles properly in all tested variants, there is a defect in electron transfer to UbQ in the S68A and R72C variants.

### Q_p_ site mutations differentially affect basal CII-driven respiration under native conditions

**Figure 3:**
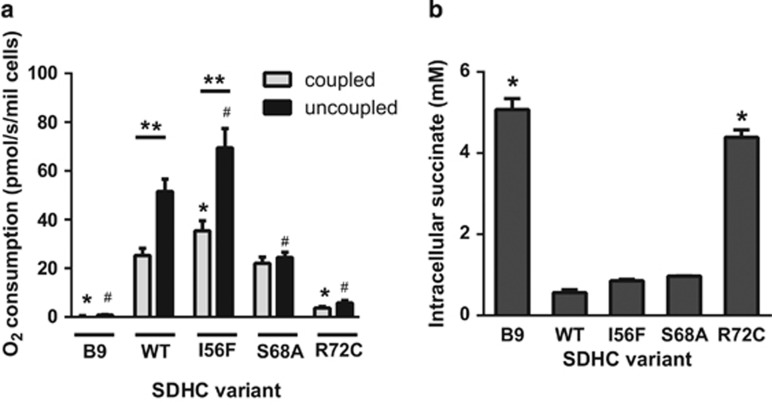
Substitutions in the Q_p_ site affect CII activity less severely in the native environment. (**a**) Oxygen consumption of digitonin-permeabilized cells respiring on 10 mM succinate in the presence of rotenone and ADP (coupled) and the maximal rate after addition of FCCP (uncoupled). While I56F and S68A variants support the respiration on succinate, B9 and R72C cells are deficient. The S68A defect is apparent only upon uncoupling. Mean±S.E.M. of 3–4 independent experiments. The symbol * indicates values significantly different from WT in the coupled state (oneway ANOVA), the symbol # values significantly different from WT in the uncoupled state (oneway ANOVA) and the symbol ** values significantly increased after FCCP addition (*t*-test). (**b**) Intracellular succinate measured by mass spectroscopy in extracts from an equal number of cells indicates functional CII in the WT, I56F and S68A. Mean±S.E.M. of two independent experiments, the symbol * indicates values significantly different from WT

As the CII activity assays described above were done on solubilized enzyme, we also examined the effect of Q_p_ site mutations on CII-mediated respiration in a more natural environment of permeabilized cells. In this set-up respiratory substrates can reach mitochondria, but the mitochondrial outer and inner membranes remain intact in their ‘native', undisturbed condition.^26^ In the presence of the CII substrate succinate, WT, S68A and, in particular, I56F cells efficiently consumed oxygen. In contrast, R72C and parental B9 cells showed little or no respiration (Figure 3a). The uncoupler carbonyl cyanide 4-(trifluoromethoxy)phenylhydrazone (FCCP) significantly increased oxygen consumption in WT and I56F, but not in S68A, R72C or B9 cells (Figure 3a). Hence, the S68A mutation only affects the reserve capacity of this mutant and may not be limiting in intact cells. In contrast, a severe defect in R72C CII substantially compromises its ability to support respiration. To confirm these findings in intact cells, we determined the steady-state levels of intracellular succinate, a proxy for CII activity.^27^ As shown in Figure 3b, succinate concentration in WT, I56F and S68A cells was low, consistent with fully functional CII. On the other hand, in B9 and R72C cells the succinate levels were considerably increased. These data demonstrate that whereas B9 and R72C cells cannot utilize succinate for respiration due to the absent or dysfunctional CII, WT, I56F and S68A cells maintain CII respiration under coupled conditions expected to occur in a physiological situation.

### Q_p_ site mutations compromise the efficacy of cell death induction by MitoVES

**Figure 4:**
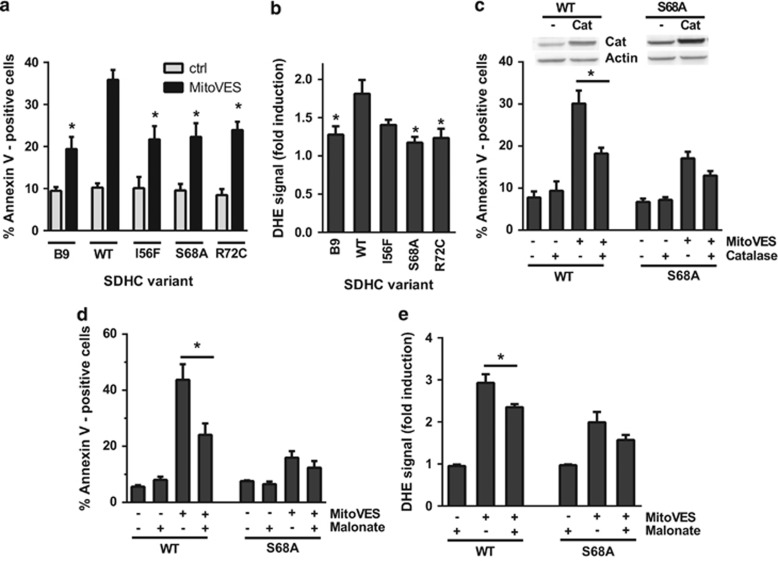
Q_p_ site substitutions lead to reduced cell death induction and ROS generation in response to MitoVES, and the induced cell death is dependent on CII-derived ROS. (**a**) Variant cell lines were exposed to 20 *μ*M MitoVES for 24 h and the percentage of annexin V-positive cells was determined by flow cytometry. *n*≥5, mean±S.E.M., * values significantly different from WT. (**b**) Variant cell lines were exposed to 2 *μ*M MitoVES for 30 min and the level of ROS was determined by dihydroethidium (DHE) staining and flow cytometry. *n*≥5, mean±S.E.M., * values significantly different from WT. (**c**) Cells were transfected with catalase-coding or control vector, exposed to 20 *μ*M MitoVES for 20 h and the percentage of annexin V-positive cells was determined by flow cytometry. *n*≥4, mean±S.E.M., * values significantly different between catalase and mock-transfected cells. Inset, catalase overexpression verified by western blot. (**d**) Cells were exposed to 30 *μ*M MitoVES for 12 h in the presence or absence of 20 mM malonate (30 min pretreatment). The percentage of annexin V-positive cells was determined by flow cytometry. *n*≥3, mean±S.E.M., * values significantly different in the presence and absence of malonate. (**e**) Cells were exposed to 5 *μ*M MitoVES for 30 min in the presence or absence of 50 mM malonate (30 min pretreatment) and the level of ROS was determined by DHE staining and flow cytometry. *n*=5, mean±S.E.M., * values significantly different in the presence and absence of malonate

Should cell death induction by the VE analog MitoVES be dependent on its binding to the Q_p_ site of CII as our previous work suggested,^16^ then the efficacy of this agent would be compromised in Q_p_ site mutants. Hence, the variant cell lines were exposed to MitoVES, a compound previously described to induce apoptosis,^17^ and the percentage of annexin V-positive cells quantified. The induced cell death displayed signs typical for apoptosis (Supplementary Figures S2A and B), and was significantly reduced in all tested variant cell lines compared with WT cells (Figure 4a). In fact, all substitutions in the Q_p_ site reduced MitoVES-induced cell death nearly to the level observed in parental B9 cells. Given the absence of assembled CII in B9 cells, this basal level of cell death must be CII independent and possibly related to the direct effect of MitoVES on cytochrome c.^28^

As ROS generation is the pivotal, early event in the cell death-initiating cascade induced by MitoVES,^16, 17^ we assessed ROS production in cultured cells upon MitoVES treatment with dihydroethidium, a fluorescent probe responsive to superoxide. Compared with WT, all Q_p_ site variant cells showed reduced ROS formation, which remained at the level similar to that of parental B9 cells (Figure 4b). In addition, catalase overexpression and co-treatment with the dicarboxylate site inhibitor malonate reduced cell death and ROS production in WT cells to the level found in the mutants (Figures 4c–e). These data indicate that the Q_p_ site mutations decrease the efficacy of MitoVES-induced ROS generation and that the induced cell death depends on CII-derived ROS.

Mitochondrial glycerophosphate dehydrogenase (GPD2) feeds electrons into the mitochondrial UbQ pool similarly to CII and can also produce ROS. It has recently been shown that alpha tocopheryl succinate (*α-*TOS), an untargeted analog of MitoVES, inhibits GPD2 activity in brown adipose tissue (BAT).^29^ However, it is unlikely that GPD2 is responsible for the observed ROS and cell death induction upon MitoVES treatment in our experimental model, as the GPD2 levels are much lower than in BAT (Supplementary Figure S2D). In addition, GPD2 inhibition by *α*-TOS decreases, rather than increases, GPD2-derived ROS^29^, and MitoVES accumulation at the inner mitochondrial membrane/matrix interface^16^ owing to the mitochondria-targeting triphenyl phosphonium group will keep it physically separated from the intermembrane space-localized GPD2. The role of the reverse electron flow from GPD2 to FAD^6^ in CII and subsequent ROS generation from that site can also be discounted, as this would be inhibited, not stimulated, by MitoVES bound to the Q_p_ site. Therefore, CII functioning in the forward manner is the likely source of ROS observed upon MitoVES treatment of the intact cells.

### Sensitivity to Q_p_ site inhibition correlates with the efficacy of cell death induction unless succinate is rapidly accumulated

**Figure 5:**
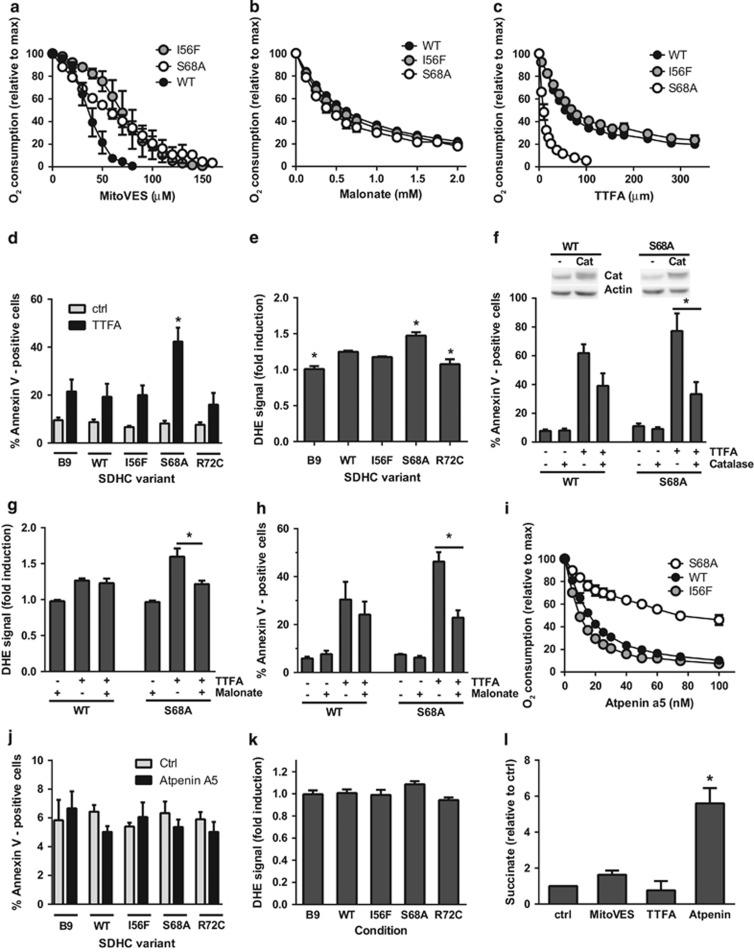
Suppression of CII-driven respiration correlates with cell death for Q_p_ site inhibitors that do not rapidly increase the succinate level. (**a**) Digitonin-permeabilized respiration-competent variant cell lines respiring on 10 mM succinate in the presence of 0.5 *μ*M rotenone and FCCP were exposed to increasing concentrations of MitoVES. The variants show reduced inhibition compared with WT. (**b**) Inhibition by the dicarboxylate-binding site inhibitor malonate in the same experimental set-up shows similar efficiency for all Q_p_ site substitutions. (**c**) Similar to **a** and **b**, but the Q_p_ site inhibitor TTFA was used. (**a**–**c**) The data represent the mean±S.E.M. of 3–4 independent experiments. (**d**) Variant cell lines were exposed to 0.5 mM TTFA for 24 h and the percentage of annexin V-positive cells was determined by flow cytometry. *n*=5, mean±S.E.M., * values significantly different from WT. (**e**) Cells were exposed to 250 *μ*M TTFA for 30 min and the level of ROS was determined by dihydroethidium (DHE) staining and flow cytometry. *n*=5, mean±S.E.M., * values significantly different from WT. (**f**) Cells were transfected with catalase-coding or control vector, exposed to 2 mM TTFA for 20 h, and the percentage of annexin V-positive cells was determined by flow cytometry. *n*=4, mean±S.E.M., * values significantly different between catalase and mock-transfected cells. Inset, catalase overexpression verified by western blot. (**g**) Cells were exposed to 250 *μ*M TTFA for 30 min in the presence or absence of 50 mM malonate (30 min pretreatment), and the level of ROS was determined by DHE staining and flow cytometry. *n*=4, mean±S.E.M., * values significantly different in the presence and absence of malonate. (**h**) Cells were exposed to 1.5 mM TTFA for 12 h in the presence or absence of 20 mM malonate (30 min pretreatment). The percentage of annexin V-positive cells was determined by flow cytometry. *n*=4, mean±S.E.M., * values significantly different in the presence and absence of malonate. (**i**) Atpenin A5-induced inhibition of respiration of permeabilized cells as described in **a**. *n*≥3, mean±S.E.M. (**j**) Cells were exposed to 1 *μ*M Atpenin A5 for 24 h and the percentage of annexin V-positive cells was determined by flow cytometry. *n*=3, mean±S.E.M. (**k**) Cells were exposed to 0.5 *μ*M Atpenin A5 for 30 min and the level of ROS was determined by DHE staining and flow cytometry. *n*=5, mean±S.E.M. (**l**) Succinate levels were determined in WT cells exposed to 20 *μ*M MitoVES, 1 mM TTFA or 1 *μ*M Atpenin A5 for 30 min. *n*=3, mean±S.E.M., * values significantly different from control

If the attenuated ROS and cell death induction described above resulted from the reduced displacement of UbQ by MitoVES at the Q_p_ site of variant CII, then these variants should display resistance to inhibition by MitoVES. For this reason, we assessed the effect of increasing concentrations of MitoVES on CII-driven respiration at high succinate (10 mM). Only respiration-competent variants, that is WT, I56F and S68A lines, were used in this experiment. As shown in Figure 5a, the efficacy of respiration inhibition was reduced for I56F and S68A variants compared with the WT cells, in direct correlation with the observed decrease in ROS levels and cell death induction (see, for example, Figures 4a and b). In contrast to MitoVES, malonate suppressed oxygen consumption similarly for all the CII variants tested (Figure 5b), confirming that the mutations introduced do not substantially affect the dicarboxylate site.

To better understand this phenomenon, we performed *in silico* molecular docking simulation of MitoVES and UbQ with the Q_p_ site of WT and variant CII. These simulations support the assumption that steric hindrance and differences in affinity can explain the less efficient inhibition by MitoVES in S68A and I56F CII (Supplementary Figures S3B and C). Since MitoVES is a relatively large molecule (Supplementary Figure S3A), we also examined the much smaller Q_p_ site inhibitor TTFA using the same methodology. Surprisingly, the highest TTFA-binding affinity was calculated for the Q_p_ site of the S68A variant (Supplementary Figure S3C). We therefore evaluated oxygen consumption in the presence of increasing concentrations of this smaller Q_p_ site inhibitor (Figure 5c) and found that while for the I56F variant the inhibition by TTFA was similar to WT cells, the S68A variant was inhibited much more efficiently. For this reason we speculated that the S68A mutation could also lead to enhanced cell death induction by TTFA, which is known to induce apoptosis.^14^ Indeed, while ROS and cell death were significantly increased in S68A cells, only limited ROS and cell death induction were observed in B9, I56F, R72C and WT cells upon TTFA treatment (Figures 5d and e). Features typical of apoptotic cell death were observed (Supplementary Figures S2A and C), and catalase overexpression or malonate treatment reduced cell death and ROS in S68A cells more than in WT cells (Figures 5f–h). These results suggest a direct correlation between the potency of Q_p_ site inhibition, CII-mediated ROS production and the extent of ensuing cell death for MitoVES as well as TTFA.

Surprisingly, the CII Q_p_ site inhibitor Atpenin A5,^24, 30^ despite efficient suppression of respiration in WT and I56F cells (Figure 5i), did not induce cell death or ROS production (Figures 5j and k). In contrast to MitoVES and TTFA, Atpenin caused rapid increase of intracellular succinate in intact cells (Figure 5l), which is incompatible with ROS generation from CII. Hence, CII inhibition results in cell death only when succinate accumulation is not too rapid and ROS can efficiently be produced.

### ROS generation in isolated mitochondria correlates with effective Q_p_ site inhibition at low succinate levels

**Figure 6:**
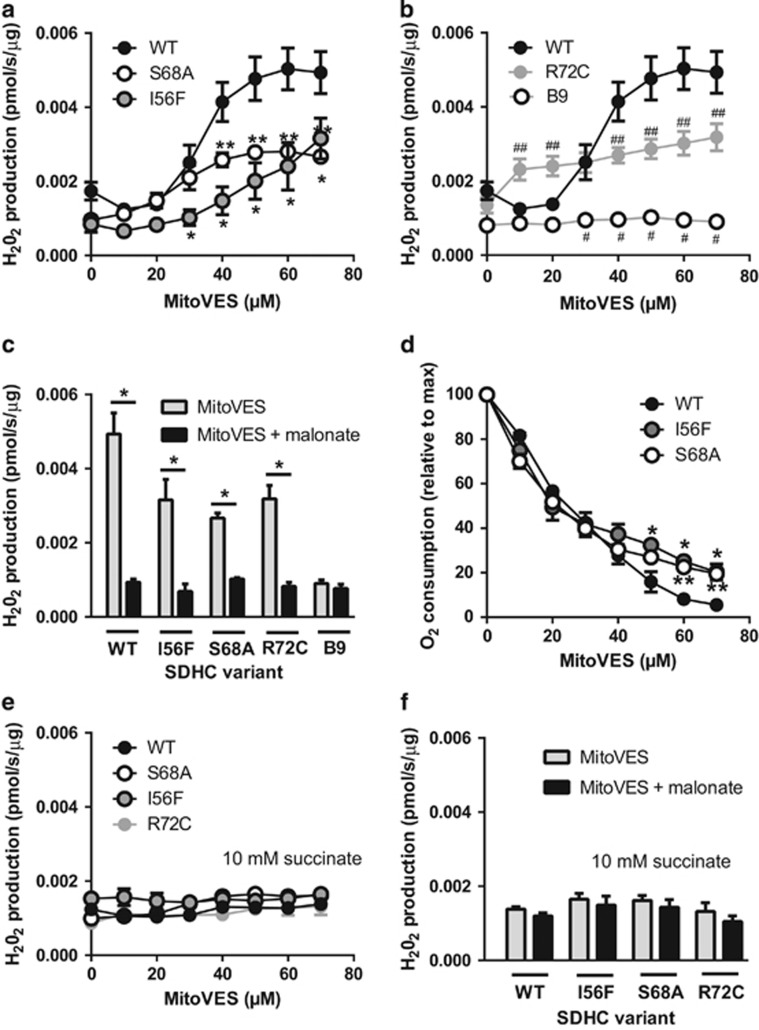
ROS induction by MitoVES correlates with CII inhibition in isolated mitochondria at low succinate. (**a** and **b**) Mitochondria isolated from variant cells respiring on 500 *μ*M succinate in an oxygraph chamber were exposed to increasing concentrations of MitoVES, and ROS production was followed in real time in the presence of Amplex Ultra Red and peroxidase. (**a**) Shows reduced ROS production for I56F and S68A variants compared with WT, (**b**) shows the same for R72C and B9 cells. The same WT data are used in **a, b**, the panels are separated for clarity only. (**c**) Under the same conditions, 5 mM malonate inhibits ROS generation by 70 *μ*M MitoVES in all cell lines except for B9 cells. (**d**) Respiratory data extracted from the experiments shown in **a** reveal that the respiration of WT cells is inhibited by MitoVES most efficiently. (**e**) ROS were measured as in **a**, **b**, but at 10 mM succinate. No increase in ROS generation was detected. (**f**) At 10 mM succinate there is no effect of 5 mM malonate on ROS at 70 *μ*M MitoVES. Data represent the mean±S.E.M. of 3–5 independent experiments. Significant differences from WT: *I56F, **S68A, ^#^B9, ^##^R72C. Panel c: * denotes a significant decrease after the addition of malonate

To establish the link between ROS production and CII inhibition, we assessed these two parameters simultaneously in isolated mitochondria by combining the Amplex Red method of ROS detection with oxygen consumption measurements. We used 0.5 mM succinate, because this concentration closely reflects its non-pathological intracellular levels (see Figure 3b) and favors direct ROS production from CII.^6, 7^ Exposure of WT cell mitochondria to increasing concentrations of MitoVES resulted in considerable stimulation of ROS production. In contrast, ROS generation was limited in S68A and I56F mitochondria (Figure 6a). A modest level of ROS production was also observed in R72C mitochondria, indicating that the low respiration rate of this mutant can still support ROS generation in response to its inhibition. As expected, no ROS increase upon MitoVES treatment was detected in mitochondria from parental B9 cells, which do not assemble CII (Figure 6b). In addition, the induction of ROS by MitoVES was malonate sensitive, confirming the involvement of CII (Figure 6c). Evaluation of oxygen consumption revealed reduced sensitivity of CII variants to inhibition compared with WT mitochondria. This effect was visible at higher concentrations of the inhibitor (Figure 6d), which, importantly, is within the concentration range where ROS induction becomes apparent (see Figure 6a). Finally, no stimulation of ROS production by MitoVES could be detected at a high succinate concentration (10 mM) for any of the variants tested (Figure 6e), which was confirmed by their insensitivity to malonate (Figure 6f).

**Figure 7:**
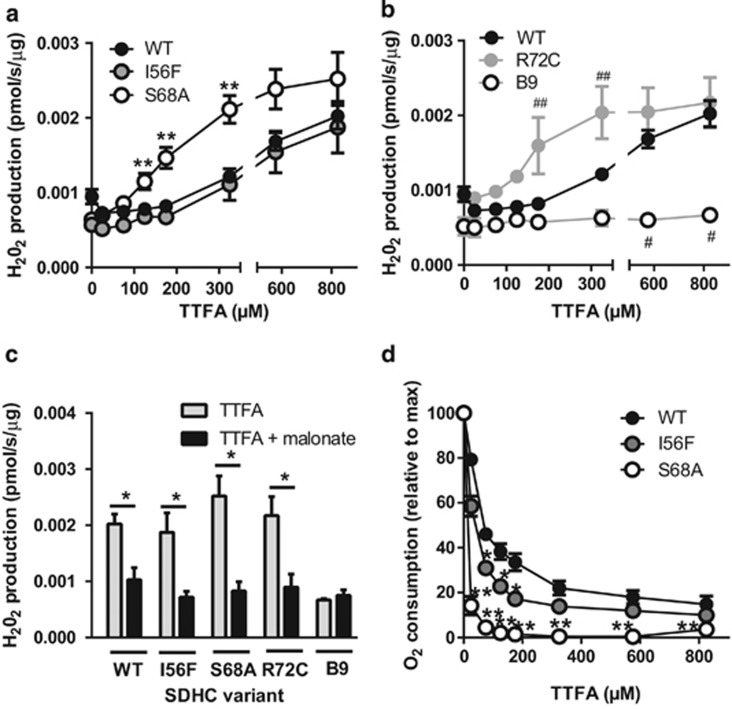
ROS induction by TTFA also correlates with CII inhibition in isolated mitochondria at low succinate levels. (**a** and **b**) Mitochondria isolated from variant cells respiring on 500 *μ*M succinate in an oxygraph chamber were exposed to increasing concentrations of TTFA, and ROS production was followed in real time in the presence of Amplex Ultra Red and peroxidase. (**a**) Shows increased ROS production for S68A variant compared with WT, (**b**) shows the higher ROS for R72C and no ROS for B9 cells. The same WT data are used in **a** and **b**, the panels are separated for clarity only. (**c**) Under the same conditions, 5 mM malonate inhibits ROS generation by 825 *μ*M TTFA in all cell lines except for B9 cells. (**d**) Respiratory data extracted from the experiments shown in **a** reveal that the respiration of S68A cells is inhibited by TTFA most efficiently. Data represent mean±S.E.M. of 4–5 independent experiments. Significant differences from WT: *I56F, **S68A, ^#^B9, ^##^R72C. **c**: * denotes a significant decrease after the addition of malonate

Compared with MitoVES, ROS induction by TTFA followed a different pattern. While for the I56F variant it was similar to WT, much more ROS were generated by S68A cell mitochondria (Figure 7a). This is in agreement with the very high sensitivity of this mutant to the inhibition by TTFA (Figure 5c). Surprisingly, induction of ROS in R72C mitochondria was also increased, which is supported by computer modeling and is expected to occur only at low succinate levels not encountered in intact R72C cells (see Discussion for details; see Figures 3b and 5e, Supplementary Figure S3C). In contrast, no ROS production was induced in B9 cell mitochondria (Figure 7b). Similarly to MitoVES, TTFA-induced ROS generation was suppressed by malonate (Figure 7c), directly implicating CII.

With non-saturating concentrations of succinate, the build-up of oxaloacetate may lead to CII inhibition at the dicarboxylate site,^6, 7, 31^ complicating the interpretation of results. Oxaloacetate accumulation is prevented in the presence of CI inhibitor rotenone, which at the same time limits reverse electron transfer to CI.^8, 32^ The inclusion of rotenone in experiments did not substantially alter the response to either MitoVES (Supplementary Figure S4) or TTFA (Supplementary Figure S5), excluding these additional factors. In summary, these results demonstrate that MitoVES and TTFA induce ROS from CII in direct proportion to their ability to achieve Q_p_ site inhibition at low, physiological succinate, which in turn correlates with the efficacy of cell death induction.

## Discussion

In the last decade it has become clear that various complexes of the mitochondrial ETC have a multifaceted role in the execution of cell death.^33, 34, 35^ CII has received particular attention as a target of experimental anticancer agents, and the inhibition of the Q_p_ site of CII was shown to induce cell death in cancer cells *in vitro* and *in vivo*.^16, 36, 37^ Clear evidence was missing, however, because the potential function of CII as an amplifier of pro-death signals originating elsewhere should also be considered.^13, 14^ In principle, it cannot be excluded that a given compound, anticipated to engage the Q_p_ site of CII, triggers a pro-death signal further upstream, followed by indirect amplification of this signal at CII. It is impossible to distinguish between these two scenarios using cellular models where CII is not assembled or is inhibited at the dicarboxylate site. Hence, to demonstrate the autonomous role of CII in cell death induction, functional CII is required.

The CII Q_p_ site variants I56F and S68A employed in this report respire on succinate similarly to WT under native conditions, yet display alterations in cell death induction upon Q_p_ site ligation with MitoVES and TTFA. The level of cell death induction directly correlates with the efficacy of inhibition of succinate-driven respiration by these agents. Accordingly, both I56F and S68A variants were relatively resistant to the inhibition by MitoVES and to cell death induced by this agent, whereas the S68A variant, which is more efficiently inhibited by TTFA, underwent proportionally higher level of cell death upon TTFA treatment. This likely stems from the altered ability of the two inhibitors to displace UbQ at the Q_p_ site of the individual variant proteins. Indeed, experimental data could be explained by different binding affinities and various degrees of steric hindrance computationally predicted for variant Q_p_ sites (Supplementary Figure S3). These observations are consistent with the direct, autonomous role of CII in cell death initiation by these agents, and cannot be reconciled with the role of CII as a mere amplifier of upstream effects originating at other sites. The engagement of cell death induction pathways unrelated to CII can be also discounted. In the latter scenarios, all variants retaining CII activity would behave similarly, which is clearly not the case. Compared with WT cells, the variant cell lines show lower response to MitoVES but higher (or similar) level of cell death upon TTFA treatment. This indicates that the mutations are not associated with nonspecific defects in cell death induction in our system, and excludes CII-unrelated ROS sources such as GPD2. Accordingly, this study establishes, for the first time, a direct connection between CII inhibition at the Q_p_ site and initiation of cell death.

**Figure 8:**
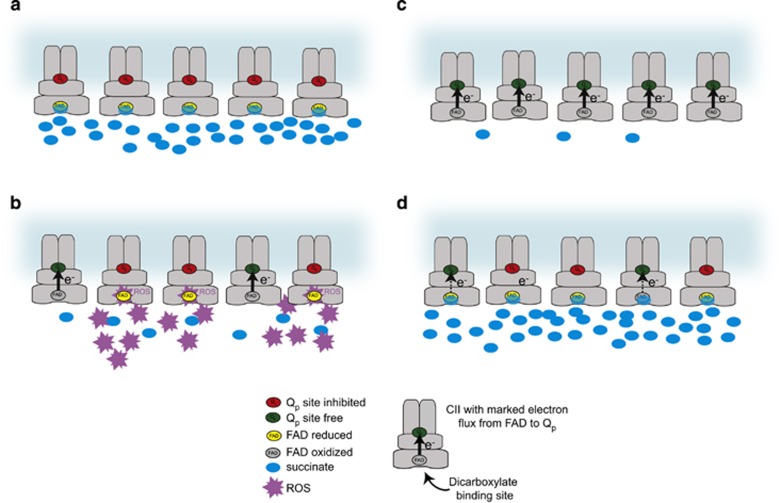
A proposed model of cell death initiation at CII explaining the regulation of ROS production from reduced FAD group in intact cells. (**a**) High-affinity Q_p_ site inhibitors, such as Atpenin A5, will immediately block most of the available Q_p_ sites in a cell and rapidly upregulate intracellular succinate. CII will be inhibited and FAD reduced, but no ROS will be produced, because succinate in the dicarboxylate site will block oxygen access. (**b**) Medium affinity inhibition such as with MitoVES or TTFA will not immediately block all the available Q_p_ sites, and some free CII will be left to keep succinate levels from rising rapidly. Because of the free dicarboxylate site, the reduced FAD in Q_p_-inhibited CII molecules will be able to produce cell death-inducing ROS. (**c**) Mutations in the Q_p_ site that do not affect CII activity will lower the ability of an inhibitor such as MitoVES to displace ubiquinone, and despite low intracellular succinate FAD will not be reduced and therefore unable to produce ROS. (**d**) Q_p_ site mutations that affect CII activity will upregulate succinate, blocking dicarboxylate site and preventing ROS generation from FAD. Additional Q_p_ site inhibition will not generate ROS under these conditions

In sub-mitochondrial particles, it has been previously established that CII can produce ROS upon Q_p_ site inhibition only when FAD is reduced and the dicarboxylate site is unoccupied.^7, 38^ In intact cells, this introduces an additional level of complexity, because the lack of enzymatically active CII will result in the accumulation of its substrate succinate due to the poor membrane permeability of this metabolite.^27^ Succinate then might block the dicarboxylate site and restrict oxygen access to FAD, attenuating ROS production.^6^ Indeed, the R72C mutation associated with low residual enzymatic activity of CII did not reduce the TTFA-dependent induction of ROS in isolated mitochondria where succinate is low (Figure 7b, Supplementary Figure S5B), but suppressed TTFA-induced ROS and cell death in intact cells where succinate is high (Figure 3b, Figures 5d and e). Similarly, Atpenin A5, a high-affinity Q_p_ site inhibitor,^24, 30^ did not induce ROS and cell death in intact cells (Figures 5j and k) in this and an unrelated^39^ study, even though it had been previously shown to efficiently generate ROS from CII in sub-mitochondrial particles, where succinate cannot accumulate.^7^ We propose that in intact cells the blockade of the dicarboxylate site of Atpenin-inhibited CII, possibly by oxaloacetate as reported^6^ or by the rapidly accumulated succinate (Figure 5l), is the likely reason for this discrepancy. This is consistent with the known behavior of CII and suggests that CII is the bona fide source of ROS in intact cells upon Q_p_ site inhibition. Furthermore, co-treatment with dicarboxylate site inhibitor malonate suppressed ROS generation and cell death upon MitoVES and TTFA administration in responsive cell lines, and MitoVES/TTFA-induced cell death was catalase sensitive (Figures 4c–e, Figures 5f–h). We therefore propose that cell death will be induced only when the Q_p_ site is inhibited in a manner that allows ROS to be generated (Figure 8). Accordingly, Q_p_ site inhibition that is too efficient or rapid, such as with Atpenin, will suppress all CII activity and reduce FAD, but at the same time block the dicarboxylate site by succinate (or other dicarboxylate), quenching ROS formation. Slower, less efficient inhibition, such as with MitoVES or TTFA, will leave some CII molecules unoccupied, slowing down succinate accumulation such that the Q_p_ site-blocked CII molecules can produce ROS and induce cell death. Reduction of Q_p_ site inhibition by mutations that do not reduce CII activity will then leave insufficient number of Q_p_ site-blocked CII molecules to generate ROS, whereas mutations that compromise CII activity will upregulate succinate, limiting the ROS production from FAD.

In conclusion, the data presented in this study provide support for the direct role of CII in cell death initiation by demonstrating a clear correlation between the efficacy of inhibition at the Q_p_ site of CII and the magnitude of cell death in respiration-proficient CII variants for Q_p_ site inhibitors that do not excessively upregulate succinate. Despite being focused on CII, our results may also be relevant for other ETC complexes, as many ETC inhibitors reported to promote cell death also modulate cell death pathways independent of the ETC. For example, the CI inhibitor rotenone destabilizes microtubules,^40, 41^ and the CII inhibitor *α*-TOS as well as the complex III inhibitor antimycin act as BH3 mimetics.^42, 43^ To our knowledge, it has never been unequivocally shown for any of these compounds that ETC inhibition is instrumental in cell death induction by correlating ETC inhibitory efficacy of a single compound at any of the ETC complexes with the extent of cell death. Hence, this report defines the Q_p_ site of CII as a suitable target for cytotoxic agents and demonstrates that ETC targeting may present a potential clinically relevant approach to cancer treatment.^44^

## Materials and Methods

### Chemicals and reagents

All chemicals and reagents were from Sigma (St. Louis, MO, USA), unless otherwise stated. MitoVES was synthesized in-house as described earlier.^16^ Atpenin A5 was from Enzo Life Sciences (Farmingdale, NY, USA).

### Cell culture

Parental cells and variant cell lines were cultured in high-glucose (4.5 g/l) DMEM medium (Lonza, Basel, Switzerland) supplemented with 10% FCS, non-essential amino acids (both Life Technologies, Carlsbad, CA, USA) and antibiotics at 37 °C and 5% CO_2_. Eahy926 cells were cultured as described in Rohlena *et al.*^18^

### Q_p_ site mutagenesis and the generation of variant cell lines

Generation of the S68A variant was described earlier.^16^ For other variants, site-directed mutagenesis of human wt SDHC cDNA was performed in the pEF-IRES-PURO expression vector using the QuickChange Lightening mutagenesis kit (Stratagene, La Jolla, CA, USA) and the following mutagenesis primers: I56F, 5′-gtcctctgtctccccactttactatctacagttgg-3′ (forward), 5′-ccaactgtagatagtaaagtggggagacagaggac-3′ (reverse), R72C, 5′-gatgtccatctgccactgtggcactggtattgc-3′ (forward), 5′-gcaataccagtgccacagtggcagatggacatc-3′ (reverse). The sequences were confirmed by DNA sequencing and used to transfect the SDHC-deficient B9 fibroblasts using the Attractene reagent (Qiagen, Hilden, Germany), followed by incubation with 2–4 *μ*g/ml puromycin for 2 weeks. Clones were analyzed for the expression of human SDHC by RT-PCR and those selected were stably transfected with pEGFP-C3-H-Ras as described with the exception of using Attractene for transfections,^36^ after which transfectants with similar level of GFP-H-Ras expression were selected. Total RNA was collected, and the presence of the variant transcript was verified by cDNA sequencing.

### Quantitative Real time-PCR

Was performed essentially as described.^16^ Primers for human SDHC detection were 5′-cacttccgtccagaccggaac-3′ (forward) and 5′-atgctgggagcctcctttcttca-3′ (reverse).

### Western blotting

Cells were lysed in RIPA buffer (150 mM NaCl, 1.0% Nonidet NP-40, 1% sodium deoxycholate, 0.1% SDS, 50 mM Tris, pH 8.0) supplemented with protease and phosphatase inhbibitors for 30 min with shaking on ice. Protein content was determined by the BCA assay (Pierce, Rockford, IL, USA). Samples were boiled for 5 min in reducing loading buffer before separation on SDS-PAGE gels. Wet blotting was used to transfer the separated samples to nitrocellulose membranes (Whatman-GE, Little Chalfont, UK). Immunoblotting was done in TBS/tween supplemented with 5% non-fat dried milk overnight at 4 ºC. Following antibodies were used: anti-H-Ras (Santa Cruz, Dallas, TX, USA, sc-520), anti-actin HRP labeled (Cell Signaling, Danvers, MA, USA, 5125), unlabeled actin (Millipore, Darmstadt, Germany, MAB1501, used for Figure 2b), anti-cleaved caspase 3 (Cell Signaling, 9664), anti-catalase (Abcam, Cambridge, UK, Ab1877), Anti-Vdac1/porin (Abcam, ab15895), anti-SDHA (Abcam, ab14715), anti-ATPase alpha subunit (Abcam, ab14748). Rabbit polyclonal antibody to GPD2 was custom prepared.^45^ HRP-conjugated secondary antibodies were used in TBS/tween with 5% non-fat dried milk for 1 h at room temperature. WB signals were quantified using the Aida 3.21 Image Analyzer software (Raytest, Straubenhardt, Germany).

### Mitochondria isolation

Mitochondrial isolation was performed according to a recently described method, with some adaptations.^46^ Cells were released by trypsin, washed in PBS, and 40–50 × 10^6^ cells were transferred to 5 ml of mitochondria isolation buffer (200 mM sucrose, 1 mM EGTA, 10 mM Tris/Mops pH 7.4). The cell suspension was homogenized by three passes through a cell homogenizer (Isobiotec, Heidelberg, Germany) set to 10 *μ*m clearance using 5 ml syringe (SGE, 5MDF-LL-GT) at 0.5 ml/min flow at 4 ºC. The homogenate was centrifuged (at 4 ºC) at 800 × *g* for 8 min, the supernatant was collected and pre-cleared at 3000 × *g* for 5 min. The final collection of mitochondrial pellet was done at 10 000 × *g* for 15 min. Protein content was determined by the BCA assay. The mitochondria were undamaged, viable and well coupled, as determined by respirometry (see below) from their reaction to the addition of ADP (about 5x increase in respiration), FCCP (substantial increase of respiration) and cytochrome c (no or very little increase in respiration).

### Blue native electrophoresis

Isolated mitochondria were solubilized in the extraction buffer (1.5 M aminocaproic acid, 50 mM Bis-Tris, 0.5M EDTA and pH 7) containing 1.3% lauryl maltoside or 8 g digitonin / g protein. Samples comprising 20–30 *μ*g of protein were then mixed with the sample buffer (0.75 M aminocaproic acid, 50 mM Bis-Tris, 0.5M EDTA, pH 7, 5% Serva-Blue G-250 and 12% glycerol) and loaded on the precast NativePAGE Novex 4–16% Bis-Tris gels (Life Technologies) and run overnight at a constant voltage of 25 V. Separated protein complexes were then transferred to the PVDF membrane (Millipore), using the Trans-Blot Turbo transfer system (Biorad, Hercules, CA, USA). CII was detected with the anti-SDHA (2E3) antibody (Abcam, AB14715-200).

### In-gel SDH activity

Lauryl maltoside (20–30 *μ*g) or digitonine solubilized mitochondria (see above) were mixed with sample buffer containing 50% glycerol with 0.1% Ponceau dye and run on the precast NativePAGE Novex 4–16% Bis-Tris gel at constant voltage of 100 V, which was raised to 500 V after the samples entered the separation gel. Deoxycholate (0.05% ) and lauryl maltoside (0.01%) were added to the cathode buffer for higher resolution as described.^47^ Gels with separated protein complexes were incubated for 30 min in assay buffer containing 20 mM sodium succinate, 0.2 mM phenazine methosulfate and 0.25% nitrotetrazolium blue in 5 mM Tris/HCl, pH 7.4. The reaction was stopped using solution of 50% methanol and 10% acetic acid and gels were immediately photographed.

### SQR activity measurement

Mitochondria (25 *μ*g) were incubated in 200 *μ*l of 25 mM phosphate potassium buffer (pH 7.4) containing 0.1% Triton X-100, 20 mM succinate, 2 *μ*M antimycine, 5 *μ*M rotenone, 10 mM sodium azide, 50 *μ*M decylubiquinone for 3–5 min in a 96-well plate. After 30 s recording of the measurement at 600 nm, 10 *μ*l of 2,6-dichlorphenol indophenol (0.015% w/v) was added and the reaction was recorded for another 2–3 min. Identical measurements were performed in the presence of 20 mM malonate and the net SQR activity was obtained by subtracting malonate-insensitive rates.

### Mitochondrial membrane potential measurements

Cells were seeded in 12-well plates a day before the experiment. On the day of experiment, one well was used to determine the total cellular protein by BCA. The rest of the cells were collected by trypsin, washed with PBS, and resuspended in Mir05 medium (see below) at 0.5 mg/ml concentration with 10 mM succinate, 2 mM malate, 10 mM glutamate, 3 mM ADP and 20 nM tetramethylrhodamine methyl ester (TMRM). Next, 60* μ*l of this cell suspension was permeabilized with 0.1 *μ*g digitonin per *μ*g protein for 5 min at room temperature, and immediately measured on LSR-II flow cytometer (Becton Dickinson, Franklin Lakes, NJ, USA) for the TMRM fluorescence signal. Finally, 0.2  *μl* of 1 mM carbonyl cyanide 3-chlorophenylhydrazone (CCCP) was added and the TMRM signal after uncoupling was assessed. The relative mitochondrial membrane potential was determined as the ratio of TMRM signal before and after the addition of CCCP (f/f_0_).

### Confocal microscopy

Live confocal images were obtained basically as described^48^ with minor modifications. The cells in complete medium in microscopy glass-bottom dishes were incubated with 250 ng/ml Hoechst 33342 nuclear stain and 10 nM TMRM for 15 min and imaged with x63 oil immersion lens at the heated stage of an SP5 confocal microscope (Leica Microsystems, Wetzlar, Germany). 2-*μ*m-thick stacks were obtained, deconvoluted with the Huygens Professional software (SVI, Hilversum, The Netherlands) and presented as maximal intensity projections.

### Determination of intracellular succinate

Cells were cultured for 24 h, washed with PBS, scrapped and extracted in 96% ethanol in a cold methanol bath. Extracts corresponding to equal number of cells were used in further analysis (10^6^ cells per 1.6 ml). After the addition of the internal standard the extracts were dried under argon stream. Afterwards, 50 *μl* of benzyl alcohol and 30 *μ*l of TMS-chloride were added to the dried samples, and the closed Eppendorf tubes were placed in the ultrasonic bath (room temperature, 45 min) and in an oven (80 °C, 45 min). A final volume of 500 *μ*l was adjusted by adding acetonitrile. Quantification of the derivatized acids was performed with an LC–ESI/MS system (Bruker Esquire 3000, Billerica, MA, USA), in positive ionization mode. The mass spectrometer was connected to a liquid chromatography system of the 1100/1200 series from Agilent Technologies (Santa Clara, CA, USA). Reversed-phase separation of the derivatives was performed on a Supelcosil 150 × 4.6 mm column with a silica-based C-18 stationary phase (5-*μ*m particle diameter). The mobile Phase A was acetonitrile and Phase B was H_2_O, 0.1% formic acid. Agilent ChemStation for LC 3D systems B01.03 was used to control the instruments and for data processing. The gradient program was 0 min 50% B, from 0 to 8 min to 5% B and at 20 min back to the initial conditions of 50% B. The injection volume was 10 *μ*l. The LC separation and the ESI settings of the Esquire instrument were optimized utilizing a dibenzyl oxalate as an internal standard. Intracellular succinate concentration was calculated using an average cell diameter of 14 *μ*m.

### Measurement of CII respiration in permeabilized cells

Cells were collected by trypsinization, washed in PBS, resuspended in Mir05 medium (0.5 mM EGTA, 3 mM MgCl_2_, 60 mM K-lactobionate, 20 mM taurine, 10 mM KH_2_PO_4_, 110 mM sucrose, 1 g/l essentially fatty acid-free BSA, 20 mM Hepes, pH 7.1 at 30 ºC) and transferred to the chamber of the Oroboros Oxygraph-2k (Oroboros Instruments, Innsbruck, Austria) for respiration measurements at 37 ºC. The chamber was closed when the oxygen signal became stable, and after recording the routine respiration on intracellular substrates the plasma membrane was permeabilized by 10 *μ*g digitonin per million cells. The CII respiration was determined in the presence of 0.5 *μ*M rotenone, 10 mM succinate, 3 mM ADP and 10 *μ*M cytochrome c. The maximal respiration in the uncoupled state was then achieved by FCCP titration in 0.5 *μ*M steps. Antimycin A (2.5 *μ*M ) was added at the end to inhibit ETC and the residual oxygen consumption after antimycin addition was subtracted from all the results to obtain the mitochondria-specific rates.

### Inhibition of CII respiration

Cells were permeabilized as above and the effect of CII inhibitors was assessed in the presence of 10 mM succinate, 3 mM ADP, 0.5 *μ*M rotenone, 10 *μ*M cytochrome c and FCCP. The inhibitors (MitoVES, TTFA, Atpenin A5 or malonate) were titrated to the chamber in regular intervals (5 min) and the rate of oxygen consumption was assessed after each addition.^49^ Solvent only was titrated into control chambers in parallel to check for nonspecific effects and cell deterioration, but the respiration rates remained virtually unaffected (<10% decrease at the end of the experiment). Respiration rates after 2.5 *μ*M antimycin A addition were subtracted to obtain the mitochondria-specific rates.

### Simultaneous measurements of ROS production and oxygen consumption in isolated mitochondria

The chambers of the Oroboros Oxygraph instrument equipped with the O2k-Fluorescence LED2-Module (Oroboros Instruments) were calibrated at 37 °C with the Budapest-modified respiration medium ( 120 mM KCl, 20 mM HEPES, 10 mM KH_2_PO_4_, 2.86 mM MgCl_2_, 0.2 mM EGTA, 0.025% BSA, pH 7). After closing the chambers, Amplex Ultra Red (Life Technologies) and peroxidase were injected (at final concentrations 5 *μ*M and 1 U/ml, respectively), followed by the addition of 200 *μ*g of isolated mitochondria, 0.5 *μ*M rotenone (where indicated), 0.5 or 10 mM succinate and 3 mM ADP. The tested inhibitors were titrated as above, and the amount of hydrogen peroxide generated was determined based on the conversion of Amplex Ultra Red to the highly fluorescent product resorufin^50^ using excitation LED 525 nm equipped with the Amplex red filter set. Oxygen consumption was recorded simultaneously in the same chamber. Malonate (5 mM) was added at the end of the titration experiment to confirm that the signal is succinate dependent. After this, hydrogen peroxide of known concentration was titrated to the chamber in several steps to calibrate the fluorescence signal. Finally, 2.5 *μ*M antimycin A was added to subtract the nonspecific respiration rates.

### ROS measurement in intact cells

The cells were seeded in 12-well culture plates and grown for 24 h. To start the experiment, tested compounds were added to the culture medium and after 15 min, dihydroethidium was added to the final concentration of 20 *μ*M. After another 15 min, the cells were harvested by trypsin and oxidized ethidium fluorescence was measured on a LSR-II flow cytometer (Becton Dickinson) and expressed as a mean fluorescence intensity.

### Cell death measurements

Cells were seeded in 12-well culture plates and grown for 24 h. After that, the tested compounds were added as indicated. The medium was collected after the required incubation time, the adherent cells were washed by PBS and harvested by trypsin. All these fractions were combined, washed by PBS and incubated with PE- or Dy647-labeled annexin V (Becton Dickinson and Apronex, Vestec, Czech Republic) in the supplied binding buffer for at least 10 min. Hoechst 33258 was added to mark the cells with ruptured cell membrane. Annexin V-positive fraction was measured by flow cytometry. The results were expressed as the percentage of annexin V-positive cells. For the catalase experiments, cells were transfected two days before the experiment with a control or catalase-containing vector (a kind gift of Dr. S Lortz)^51^ using the Fugene transfection reagent (Promega, Fitchburg, WI, USA) according to manufacturer's instruction.

### Computer modeling

The structure of WT human mitochondrial complex II was obtained as a homology model based on the highly homologous template of the porcine CII^3^ (pdb id 1zoy, sequence identity 95, 96, 92, and 88% for SDHA to SDHD) using the Modeller suite of programs.^52^ The single point SDHC mutations (I56F, S68A, R72C) were then introduced using the FoldX program,^53^ which was also used to optimize the side chain rotamers within the WT as well as mutated structures. All the structures were further subjected to a short (10 ns, implicit solvation) molecular dynamics (MDs) run in order to relax the potentially non-equilibrium structures. The MD was prepared and performed using the GPU version of the GROMACS suite of programs^54^ as implemented in the OpenMM Zephyr package.^55^ Average structures from second half of the simulations were further used for the docking study. The docking study of the MitoVES, TTFA and ubiquinone ligands/inhibitors to a series of mutated mitochondrial complex II structures was performed using the autodock suite of programs.^56^ The ligands were docked into the homology model of human CII and its single point mutants using Python Molecular Viewer version 1.5.6rc3.^57^ Each ligand was allowed to sample docking poses in a box (70 × 70 × 70 grid points with 0.375 Å spacing) centered at the level of the ubiquinone binding site (based on the crystal structure). The side chains of residues surrounding the binding site were considered flexible. A series of four separate “local search” runs of 50 cycles each was performed and the results were combined to find the most stable poses.

### Statistical analysis

Statistical analysis was performed using GraphPad Prism 6 software (GraphPad Software, La Jolla, CA, USA). Statistical significance was determined by oneway ANOVA followed by Dunnett's post test. For pair-wise comparisons (Figures 3a and 4c–e, Figures 5f–h, Figures 6c and 7c,Supplementary Figures S4C and S5C), we used unpaired *t*-test. *P*≤0.05 was considered statistically significant. The value of *n* indicates the number of independent experiments.

## Abbreviations

*α-*TOS: alpha tocopheryl succinate
CII: respiratory complex II
SDH: succinate dehydrogenase
CCCP: carbonyl cyanide 3-chlorophenylhydrazone
ETC: electron transport chain
FAD: flavin adenine dinucleotide
FCCP: carbonyl cyanide 4-(trifluoromethoxy)phenylhydrazone
GFP: green fluorescence protein
GPD2: mitochondrial glycerophosphate dehydrogenase
MitoVES: mitochondrially targeted vitamine E succinate
Q_p_ site: proximal ubiquinone binding site
ROS: reactive oxygen species
SDHC: CII subunit C
SQR: succinate-ubiquinone reductase
TCA: tricarboxylic acid
TTFA: thenoyltrifluoroacetone
TMRM: tetramethylrhodamine methyl ester
UbQ: ubiquinone
VE: vitamin E
