# Supplementary figures and images for: Ubiquinone-binding site mutagenesis reveals the role of mitochondrial complex II in cell death initiation
Source: Cell Death Dis. 2015 May 7;6(5):e1749–. doi: 10.1038/cddis.2015.110 (PMC4669690; doi:10.1038/cddis.2015.110)

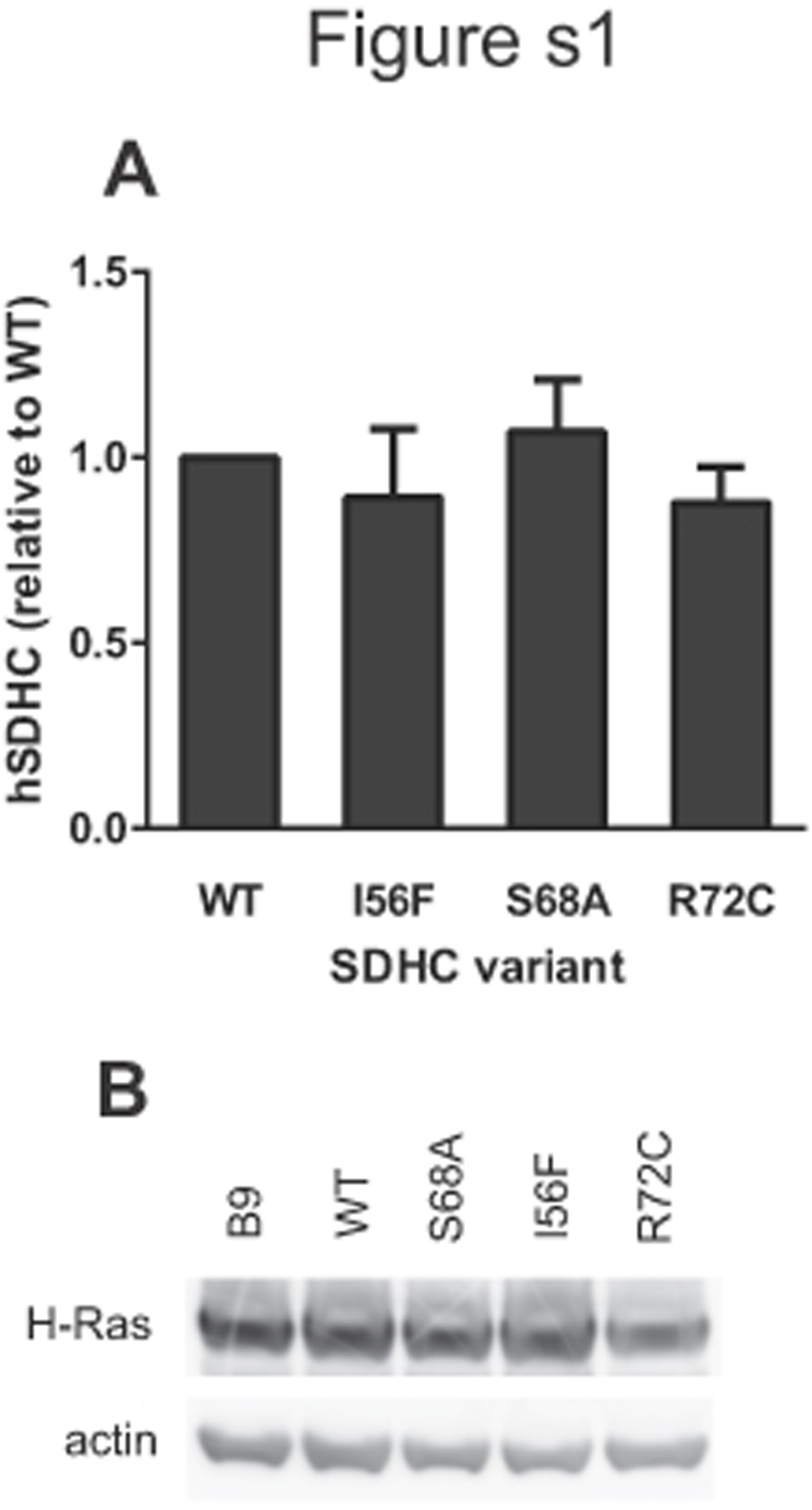

Supplement: Supplementary Figure 1 [file cddis2015110x1.tif]

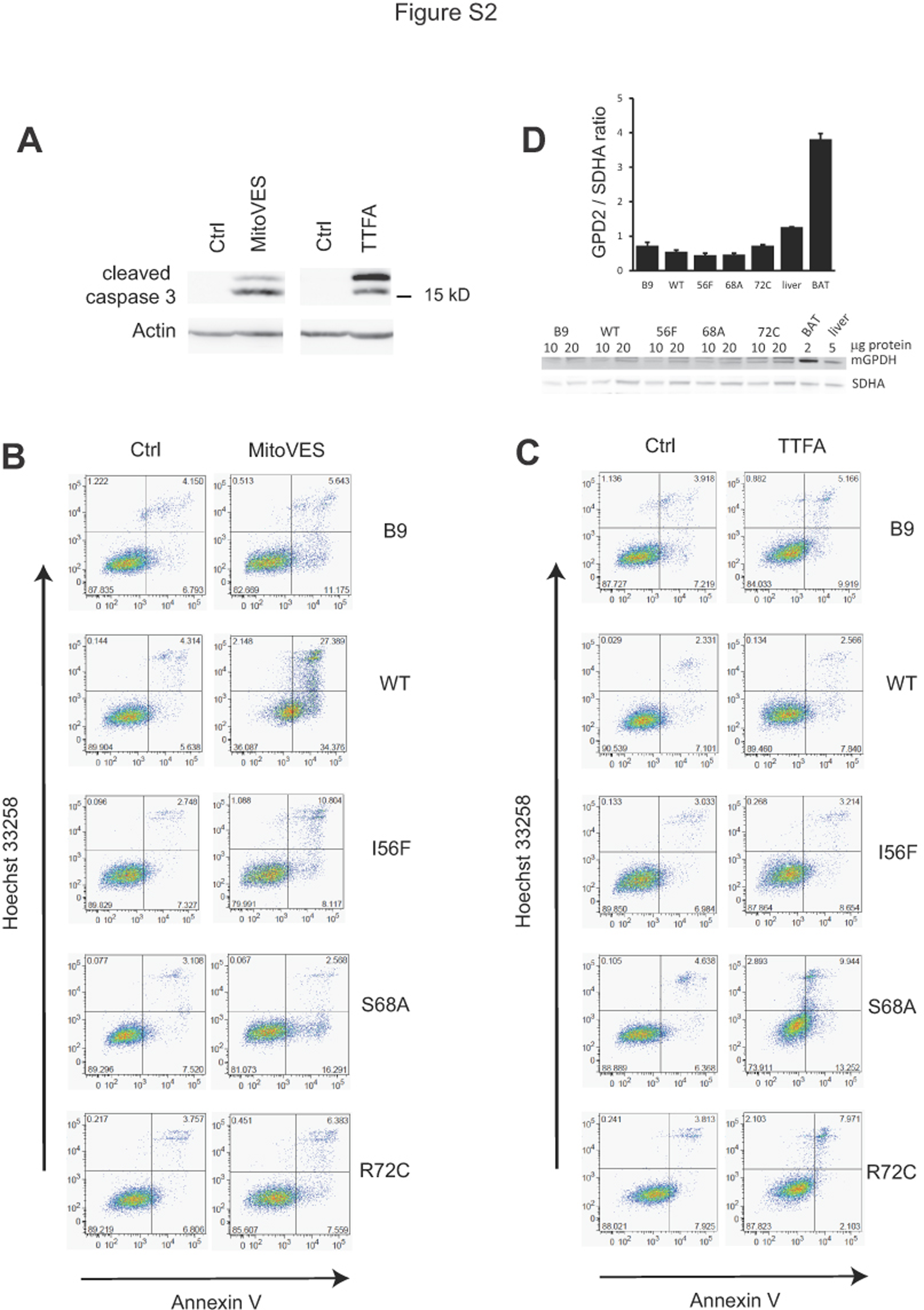

Supplement: Supplementary Figure 2 [file cddis2015110x2.tif]

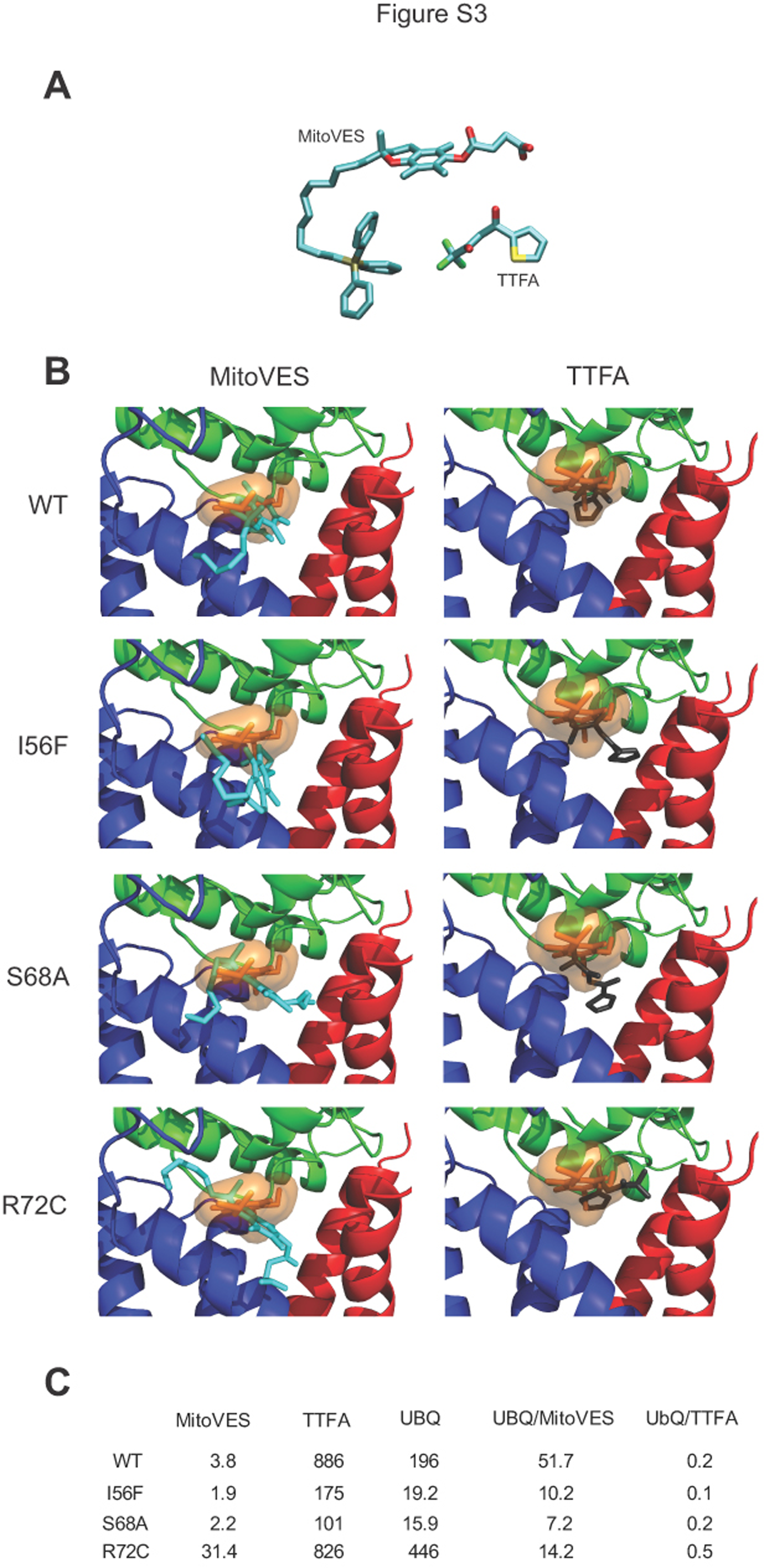

Supplement: Supplementary Figure 3 [file cddis2015110x3.tif]

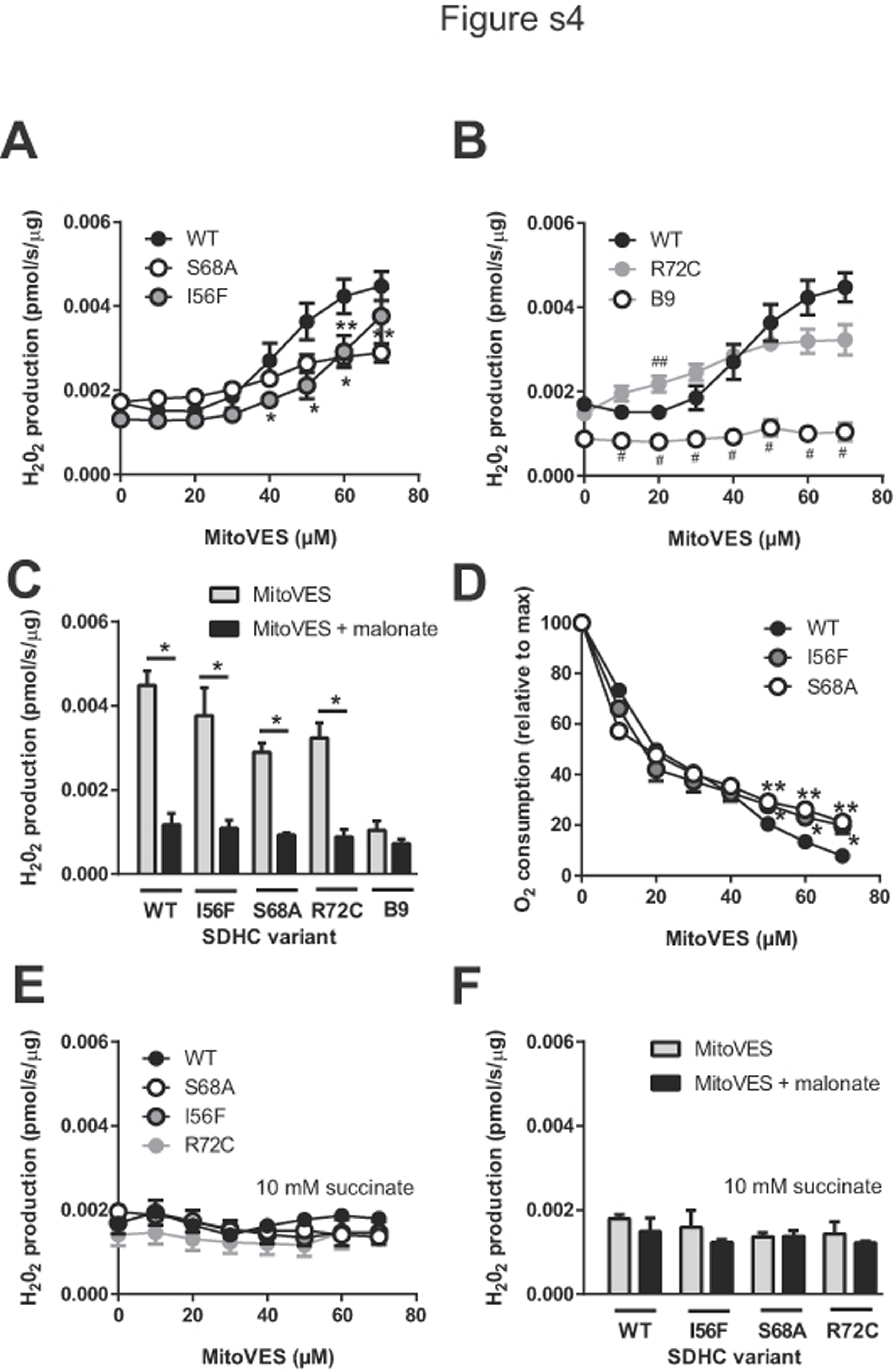

Supplement: Supplementary Figure 4 [file cddis2015110x4.tif]

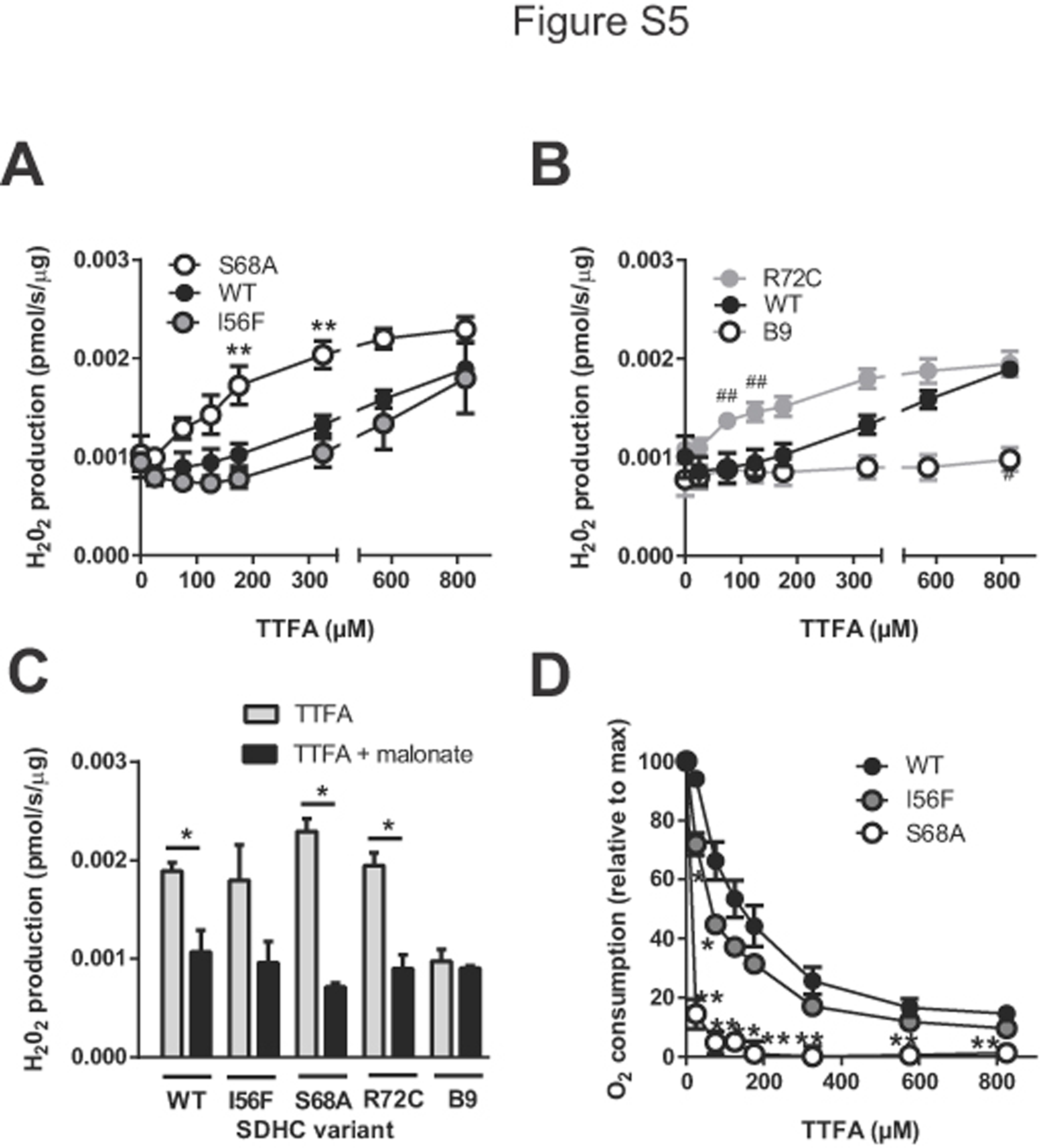

Supplement: Supplementary Figure 5 [file cddis2015110x5.tif]
