# Supplementary material for: Ubiquinone-binding site mutagenesis reveals the role of mitochondrial complex II in cell death initiation
Source: Cell Death Dis. 2015 May 7;6(5):e1749–. doi: 10.1038/cddis.2015.110 (PMC4669690; doi:10.1038/cddis.2015.110)
Supplement: Supplementary Figure Legends [file cddis2015110x6.docx]

**Supplementary figures legends**

**Figure S1.** Human SDHC and H-RAS-GFP expression in selected clones. (a) Human SDHC mRNA expression in the selected clones was measured by qPCR. Data are from at least 3 independent measurements. (b) H-RAS-GFP expression measured by western blot using H-RAS-directed antibody. Representative experiment is shown. The determination was done at least 4 times with equivalent results.

**Figure S2.** Cell death induced by CII inhibitors MitoVES and TTFA displays signs consistent with apoptosis (a) Eahy926 cells were exposed to 20 µM MitoVES or 1.5 mM TTFA for 17 h, and caspase 3 activation was determined by western blotting. These cells were used because the antibody did not reliably work in our hamster cell lines. (b) CII-variant cells exposed to 20 µM MitoVES for 24 hours were stained with annexin V and Hoechst 33258 and examined by flow cytometry. Cells staining solely for annexin V are located in the lower right quadrant. (c) The same as b, but cells were incubated with 0.5 mM TTFA. Representative images are shown. (d) GPD2 (mGPDH) protein expression in CII-variant cells used in this study. GPD2 was determined by western blotting and its expression level was assessed relatively to the content of SDHA. The quantification is based on 3 biological replicates ±SEM. A representative blot is also shown. BAT, brown adipose tissue.

**Figure S3**. Computer modelling of MitoVES and TTFA interaction with WT and variant Q_p_ sites of CII. (a) Size comparison of MitoVES and TTFA. (b) Molecular docking of MitoVES (cyan, left panels) and TTFA (dark grey, right panels) to the Q_p_ site in variant proteins in relation to ubiquinone (orange) was performed as described in ‘materials and methods’. Color coding of CII subunits is identical to Fig. 1. Humanized porcine structure^3^ was used for computations. For MitoVES, the results show a substantial steric clash with ubiquinone in WT and R72C, but much more limited one in I56F. In case of TTFA, S68A shows deepest docking of the inhibitor, which could possibly lead to efficient interference with electron transfer. (c) Calculated affinities (in µM) for binding of MitoVES, TTFA and ubiquinone (UBQ) to the Q_p_ site of variant proteins. Surprisingly, relatively little difference in binding to individual variants was obtained for MitoVES, while in case of TTFA the highest affinity was calculated for S68A. Predictions for ubiquinone are: lower affinity for R72C, and increased affinity for I56F and S68A. The affinity ratio between ubiquinone and MitoVES (UBQ/MitoVES) or TTFA (UBQ/TTFA) should give a rough estimate (based on calculated affinity only and neglecting any other factors) of ubiquinone displacement by the inhibitor (the higher the number, the better ability to displace ubiquinone).

**Figure S4** ROS induction by MitoVES correlates with CII inhibition in isolated mitochondria at low succinate in the presence of rotenone (a-b) Mitochondria isolated from variant cell lines respiring on 500 µM succinate in the presence of 0.5 µM rotenone in an oxygraph chamber were exposed to increasing concentrations of MitoVES, and ROS production was followed by Amplex Ultra Red / peroxidase. (a) shows reduced ROS production for I56F and S68A variants compared to WT, (b) shows the same for R72C and B9 cells. The same WT data are used in a-b, the panels are separated for clarity only. (c) Under the same conditions, 5 mM malonate inhibits ROS generation by 70 µM MitoVES in all cell lines except for B9 cells. (d) Respiratory data extracted from the experiments shown in a reveal that the respiration of WT cells is inhibited by MitoVES most efficiently. (e) ROS were measured as in a-b, but at 10 mM succinate. No increase in ROS generation was detected. (f) At 10 mM succinate there is no effect of 5 mM malonate on ROS at 70 µM MitoVES. Data represent mean ± SEM of 4-6 independent experiments. Significant differences from WT: * I56F, ** S68A, # B9, ## R72C. Panel c: the symbol * denotes a significant decrease after the addition of malonate.

**Figure S5.** ROS induction by TTFA correlates with CII inhibition in isolated mitochondria at low succinate in the presence of rotenone. (a-b) Mitochondria isolated from variant sub-lines respiring on 500 µM succinate in the presence of 0.5 µM rotenone in an oxygraph chamber were exposed to increasing concentrations of TTFA, and ROS production was followed by Amplex Ultra Red / peroxidase. (a) shows increased ROS production for S68A variant compared to WT, (b) the higher ROS for R72C and low for B9 cells. The same WT data are used in a-b, the panels are separated for clarity only. (c) Under the same conditions, 5 mM malonate inhibits ROS generation by 825 µM TTFA in all cell lines except for B9 cells. (d) Respiratory data extracted from the experiments shown in a reveal that the respiration of S68A cells is inhibited by TTFA most efficiently. Data represent mean ± SEM of 4-6 independent experiments. Significant differences from WT: * I56F, ** S68A, # B9, ## R72C. Panel c: the symbol * denotes a significant decrease after the addition of malonate.
